# Dietary Behaviour Pattern and Physical Activity in Overweight and Obese Egyptian Mothers: Relationships with Their Children’s Body Mass Index

**DOI:** 10.3889/oamjms.2016.095

**Published:** 2016-09-01

**Authors:** Nayera E. Hassan, Saneya Wahba, Inas R. El-Alameey, Sahar A. El-Masry, Mones M. AbuShady, Enas R. Abdel Hameed, Tarek S. Ibrahim, Samia Boseila, (Affiliation ID: 60014618)

**Affiliations:** 1 *Biological Anthropology Department, Medical Division, National Research Centre, Giza, Egypt*; 2 *Child Health Department, Medical Division, National Research Centre, Giza, Egypt*

**Keywords:** Overweight and Obese, mothers, Body Mass Index, dietary Behaviour, physical Activity

## Abstract

**BACKGROUND:**

Obesity and related morbidity increase in Egyptian women and their children. A better understanding of dietary and activity patterns is needed to reduce obesity prevalence.

**AIM:**

The present study aimed to assess dietary patterns and physical activity in Egyptian overweight and obese mothers and to explore its relationships with their children’s body mass index (BMI).

**SUBJECTS AND METHODS:**

This descriptive case-control study was conducted at the National Research Center. The study included a sample of 64 overweight and obese mothers and 75 children, compared with apparently healthy non-obese mothers and their children of matched age and social class. Tested questionnaires were used to collect information of the studied subjects.

**RESULTS:**

A statistically significantly higher incidence of unemployment, large family size was observed in overweight & obese women compared to controls (P < 0.05). Those women who consumed vegetables more than 3 times a week were less likely to be overweight or obese (P < 0.05). No significant association were detected between mothers’ physical activity, dietary behaviour variables and children’s BMI except for consuming beverages with added sugar (95%CI = 0.074-0.985, P<0.05).

**CONCLUSION:**

Improper dietary patterns, nonworking mothers and big family size are associated with obesity among Egyptian women. Emphasis should be given to increasing physical activity and encourage healthier diets among Egyptian mothers and their children.

## Introduction

Obesity has become an epidemic worldwide problem affecting both developed and developing countries [1]. The prevalence of overweight and obesity in the Arab countries has reached an alarming level ranging between 50% and 70% for women in the 21st century [2] which necessitates urgent action. In Egypt, the prevalence of obesity has increased markedly with the changing food habits and the increasingly sedentary lifestyles, with nearly 70 percent of the Egyptian adult population being obese [3]. According to the most recent World Health Organization statistics, Egypt occupies the 14th place in obesity worldwide [4]. Childhood obesity is currently around one in every ten is obese. According to CDC, the percentage of childhood obesity in the 1980’s jumped from 7 percent to 17 percent (12.5 million) in 2010 [5].

Several risk factors have been cited as potential causes for obesity in mothers’ and young children, including socioeconomic status, lack of physical activity [6], changes in eating habits such as skipping breakfast [7], consumption of fast foods rich in fat, sugar and salt, with a decrease in the intake of whole grains, legumes, fruits and vegetables or excessive eating [8, 9]. Obesity has led to an increase in the prevalence of chronic non-communicable diseases as type 2 Diabetes, and cardiovascular disease [10]. All of these factors are being the main causes of morbidity, mortality, and disability in Arab countries, and causing more than 50% of total deaths [11]. Hence, our study aim was to identify the relationships of specific behaviors (dietary patterns and physical activity) on body mass index (BMI) of a sample of Egyptian overweight and obese mothers compared to controls and to study the effect of these behaviors, maternal BMI, socioeconomic factors, family structure on their children’s BMI.

## Subjects and Methods

### Design and Sample

This descriptive case-control study was conducted at the National Research Centre (NRC), Giza, during the period from April 2014 to March 2015. The study included 64 overweight & obese mothers and 75 children compared with apparently healthy non-obese mothers and their children of matched age and social class. The mother’s age was ranging from 24-57 years (mean age, 39.38 ± 8.51 years). All studied women provided signed written consent form of the Medical Ethical Committee of NRC to participate in assessments. A thorough history and general examination of mothers and their children were done to exclude forms of obesity other than exogenous dietetic obesity.

### Methods

#### Data Collection

Data were collected through the pretested questionnaire after modification of the same items to fit the Egyptian setting. Data collectors were conducted and supervised by a senior dietitian. Dietary patterns and physical activity were estimated as times per week according to Giovannucci et al, [12].

#### Anthropometric measurements

Anthropometric measurements including weight and height were assessed for all the studied subjects at the National Research Centre. Body weight was measured to the nearest 0.1kg by a standard clinical balance. Standing body height was measured, to the nearest 0.1 cm by using Holtain Stadiometer. The scales were recalibrated after each measurement following the recommendations of the International Biological Program [13]. Body mass index (BMI) was calculated by dividing weight by height squared (kg/m^2), then we applied the cutoff points recommended by World Health Organization (WHO) reference as follows: Overweight (BMI = 25.00 - 29.99), obesity (BMI = ≥ 30.00) while those with BMI > 18-24.9 Kg/m^2 were considered normal [14].

### Data statistical analyses

Statistical analyses were performed using the SPSS statistical package software for Windows version 21 (SSPS Inc, Chicago, USA). Results were expressed as the mean ± SD, frequencies and percentages. Multiple logistic regression analysis was done to detect predictors of obesity in children. Odds ratios and 95% confidence intervals were calculated for the association between different variables and obesity in mothers and children. A p-value < 0.05 was considered significant and p < 0.005 was considered highly significant.

## Results

The study included 64 overweight and obese women (BMI > 25), with age ranging from 24-57 years (mean age, 39.38 ± 8.51 years), and their mean BMI was 35.18 ± 5.40. The age of 75 children ranged from 6-19 years and their mean BMI was 17.62 ± 8.77. Table 1 shows age and anthropometric measures of the all studied sample.

**Table 1:** Age and anthropometric measures of overweight and obese mothers and their children

Variables		Age (yrs)	Weight (kgs)	Height (cm)	BMI
Mothers	Mean ± SD	39.38 ± 8.51	88.14 ± 15.78	158.03 ± 8.06	35.18 ± 5.40
	Range	(24-57)	(55-159)	(148-195)	(25.2-50.4)
Children	Mean ± SD	10.46 ± 4.17	36.70 ± 28.61	136.67 ± 19.24	17.62 ± 8.77
	Range	(6-19)	(12.2-95.4)	(121-169)	(8.2-33.40)

Regarding the education levels of the participants, almost 83.3% have low educational levels; (20%) were illiterate, (63.3%) were up to secondary educated, while (16.7%) were university educated. About 73.3% of overweight & obese women in the studied sample are not working, while 26.7% are working. More than half of the overweight & obese sample (58.8%) had a large family size between 4 and 10 family members (adults and children) in the home. As regards unemployment, large family size, there was a statistically significantly higher incidence among overweight & obese women compared to controls (P < 0.05), while no significant relationship with their levels of education (p > 0.05), as shown in Table 2.

**Table 2:** Comparison of socio-demographic characteristics of overweight and obese women and control groups

Variables		Control group	Overweight & Obese group	P- value
Women occupation	- Non working - Worker, employee	14.3% 85.7% 0%	73.3% 26.7% 20.0%	0.002**
Women education levels	- Illiterate - Up to secondary - University educated	85.7% 14.4%	63.3% 16.7%	0.387
Family size	4 or less members >4 members	83.3% 16.7%	41.2% 58.8%	0.048*

* Significant difference at *P* < 0.05;

** highly significant difference at P < 0.005.

***Consumption of fast fried food:*** The frequency of consuming of fast fried food prepared outside the home was a common practice in 53.9 % of the studied women, at least 1-3 times per week by (40.1%) versus 4-7 times per week by (13.8%), and 44.6% did not consume any fast fried food.

***Sugary Beverages:*** The intake of sugar-sweetened beverages in the form of canned fruit juice, carbonated beverages, and hot drinks (tea, coffee, and herbal tea) with added sugar was high among the studied women (95.9 %).

***Vegetables and fruits*** were consumed at least 1-3 times weekly by 64.6% and 44.6% of the sample and 4-7 times weekly by 32.3%% and 50.9%of sample respectively.

***The breakfast meal*** was skipped by 16.9% of the studied women, while 77 % used to have breakfast; the majority of them (58.5 %) had breakfast 4-7 times weekly and the remaining 18.5 % had their breakfast at least, 1-3 times per week.

***Concerning physical activity:*** Most (76.9%) of mothers stated that, they did not practice any sort of physical activity. Walking was the main physical activity performed where 9.2% used to walk 1- 3 weekly and the same percentage 9.2% walked 4-7 times/week (Table 3).

**Table 3:** Dietary patterns and physical activity per week in the studied overweight and obese women

Variables	Dietary patterns and physical activity per week		
	4-7 times	1-3 times	0- time
1-Fast food	44.6%	40.1%	13.8%
2-Beverage with sugar	3.2%	42.9%	53.0%
3-Vegetables	0%	64.6%	32.3%
4-Fruits	0%	44.6%	50.9%
5-Breakfast meal	16.9%	18.5%	58.5%
6-Physical activity	76.9%	9.2%	9.2%

Comparing the weekly dietary behaviour between the studied samples revealed a higher percentage of fast food consumption in overweight & obese mothers (15.7% vs. 42.9%) respectively with no significant differences). Having breakfast and consumption of sugar-sweetened beverages was a common practice in the overweight & obese group, with no significant difference when compared to the control group (P > 0.05). There was no statistical difference between overweight & obese mothers as regards physical activity, both groups being mostly inactive (Table 4).

**Table 4:** Comparison between cases and controls as regard to practising physical activity and some diet consumption

Variables	Behavior during last week	Control group	Overweight & Obese group	P- value
Physical activity / week	No Yes	85.7% 14.3%	81.4% 18.6%	0.77
Dietary patterns					
- Fast food / week	No Yes	57.1% 42.9%	48.3% 51.7%	0.659
- Beverage with added sugar / week	No Yes	0% 100%	3.4% 96.6%	0.62
- Breakfast / week	No Yes	14.3% 85.7%	17.2% 82.8%	0.844

*Significant difference at *P* < 0.05.

Table 5 showed that women who consumed vegetables more than 3 times per week were less likely to be overweight or obese (OR = 0.176, 95% CI 0.031-0.991, P < 0.05).

**Table 5:** Association between frequency of vegetables and fruits consumption and mother’s overweight and obesity women

Variables		Non-obese women	Overweight & Obese women	Pearson Chi-Square	Odds ratio	95% CI	P value
Vegetables consumption per week	1-3 times	28.6%	69.5%	4.61	00.176	0.031-0.991	0.032*
	4-7 times	71.4%	30.5%				
Fruits Consumption per week	1-3 times	57.1%	49.2%	0.160	10.39	0.284-6.707	0.690
	4-7 times	42.9%	50.8%				

* Significant difference at P < 0.05.

The regression analysis showed a statistically significant positive association between children’s BMI and consumption of beverages with added sugar by their mothers (95% CI 0.074-0.985, P < 0.05). The other variables as maternal BMI, education, occupation, family size, room number, physical activity, and other dietary behaviour variables were not significant predictors of children’s BMI as shown in Table 6.

**Table 6:** Association between children BMI category and mother’s BMI, socio-demographic characteristics, dietary behaviour, and physical activity (Multiple regression analysis)

Variables	B	Odds ratio	95% CI	P- value
Maternal education	-0.209	0.811	0.219-3.0	0.755
Maternal occupation	-0.076	0.927	0.125-6.8	0.941
Family size	0.1	1.1	0.256-4.7	0.893
Number of rooms	-0.105	0.9	0.219-3.6	0.884
Maternal BMI	-0.804	0.448	0.37-5.3	0.526
Beverages / week	1.331	1.259	0.074-0.985	0.047*
Vegetables / week	-0.135	0.873	0.602-1.267	0.476
Fruits/ week	-0.214	0.807	0.514-1.267	0.352
Breakfast / week	0.075	1.078	0.77-1.51	0.660
Physical activity	-0.439	0.645	0.25-1.60	0.346

* Significant difference at P < 0.05.

## Discussion

Obesity is one of the most prevalent nutrition disorders with increasing trend among people of all ages all over the world creating a health and economic burden on their government services [1]. Most of the Arab countries had a major change in their lifestyle pattern including food consumption and nutrient intake of high-caloric during last decades [15]. A sedentary lifestyle has lead to the significant prevalence of obesity among Egyptian populations [16]. Hence, the aim of this study was to identify the relationship of specific behaviors (dietary patterns and physical activity) that are known to affect energy intake with the BMI among a sample of overweight and obese Egyptian mothers and to explore the possible association between maternal BMI, socioeconomic factors, family structure and the BMI of their children.

Our study included 64 overweight and obese women, with an age ranging from 24-57 years (mean age, 39.38 ± 8.51 years) with a mean BMI of 33.98 ± 6.28. Seventy-five children with an age ranged from 6-19 years and their mean BMI was 17.62 ± 8.77.

Education plays a role in obesity prevalence, where illiteracy was associated with obesity in the Gulf countries [17]. In the present study, economic status was measured by the education level, occupation, and family size of overweight and obese women compared to that of the controls. A large family size and low occupation percentages were common among overweight and obese women. About 73.3% of overweight and obese women were not working while 85.7% of the control group were employed (p = 0.002). As regards education of the overweight and obese women, (20%) were illiterate, (63.3%) were educated up to secondary school level, while only (16.7%) had a university degree, the difference with the control group was statistically not significant although none of the mothers of the control group was illiterate. Our findings are in agreement with studies from Gulf countries, and Colorado that has shown a strong relationship between obesity prevalence and low educational level, unemployment, poverty, and low-income [17, 18].

Maternal behaviours influence their children’s behaviours directly and the shared home environment also provides access to foods among them, such as eating fast food meals together [19]. In our study, more than 50% (53.9%) of overweight and obese women consumed fast food 1-7 times per week. Fast foods are considered a high source of total energy, total fat, saturated fat, and cholesterol. Higher frequency of fast-food consumption with added sugars has been reported in persons of lower socioeconomic status among obese women in African Americans, and Arab countries [20-22].

Consuming a diet high in fruits and vegetables is associated with lower risks of numerous chronic diseases. Fruits and vegetables in their natural state have high water and fibre content and thus are low in calories and energy density which may help people feel full with a greater quantity of food at a meal while consuming fewer calories [23].

Vegetables were consumed almost on a daily basis by 71.4% of the non-obese mothers with a significantly higher percentage than the rate of consumption of the obese and overweight mothers (p < 0.005). In our study, vegetable consumption was the only strong protector associated with controlling body mass index against obesity with a significance of less than 0.05. No significant difference in the frequency of fruit consumption was detected between the 2 groups (P > 0.05). Our results were similar to some other studies in Australia and Spain that have reported low consumption of vegetables in overweight and obese women [23, 24]. Data from WHO (Regional Office in Cairo) indicate that 79% to 96% of adults in 6 Arab countries (Egypt, Jordan, Iraq, Kuwait, Saudi Arabia, and Syria) eat less fruit and vegetables per day [11].

Skipping breakfast may be related to obesity, as individuals who do not eat early in the morning, may feel hungry later on and then may consume a greater number of calories during the evening hours than individuals who eat consistently throughout the day [25, 26]. In our study, 77 % of the overweight and obese women had their breakfast meal and it was skipped by only 16.9%, but most of the Egyptian housewives tend to have late breakfast. The majority of the participants had irregular meals with two main meals per day.

Regular consumption of sugar-sweetened beverages, particularly carbonated soft drinks, contributes to the epidemic of overweight and obesity [27, 28]. In our study, there was no significant difference between overweight & obese and non-obese women concerning the intake of sugar-sweetened beverages in the form of canned fruit juice, carbonated beverages, and tea with added sugar. This is not in agreement with Rasheed et al., [29] who found that regular drinking of soft drinks was significantly more common among Saudi obese women than non-obese women. On the other hand, consumption of beverages with added sugar by the mothers in our study was the only significant predictor for children’s BMI, most probably due to easy access to these beverages kept in the house.

Lack of physical activity in adults of 7 Arab countries (Egypt, Iraq, Jordan, Kuwait, Saudi Arabia, Sudan, and Syria) ranged from 33% to 86% according to WHO statistics [11]. The increase in the number of hours spent watching television and dependence on modern means of transport and the decreasing physical activity leads to the sedentary lifestyle in Arab societies [30].

Analysis of physical activity pattern in our study has shown that more than 3/4 of the mothers (76.9%) had a sedentary life pattern. Walking and housework were almost the only activities that the participants were engaged in. Walking was practised by 9.2% of the participants for 1-3 times/week while the same percentage (9.2%) walked 4-7 times weekly.

The problem of childhood obesity is not only what the children eat, but also their consumption patterns which mean where, when, and how much (amount) of food does a child eat [31, 32]. Our results showed a statistically positive association between children’s BMI and consumption of beverages with added sugar by the mothers (95%CI 0.074-0.985, P < 0.05), other variables as physical activity, dietary behaviour were not significant predictors of children’s BMI. Since parents are influential in the development of health behaviours of their children; efforts to reduce obesity should take into consideration the child’s behaviours and weight status within the family [33].

In conclusion, nonworking mothers, large family size, and higher frequency of fast-food consumption with added sugars are associated with obesity among the studied Egyptian mothers. We recommend the encouragement of consuming healthy diets and the promotion of physical activity among Egyptian mothers, and their children.

*Limitations:* The present study had several notable limitations including the small sample size, in addition to the wide age range of children which made it difficult for more statistics.
